# Risk factors for intraoperative endplate injury during minimally-invasive lateral lumbar interbody fusion

**DOI:** 10.1038/s41598-021-99751-6

**Published:** 2021-10-11

**Authors:** Young-Hoon Kim, Kee-Yong Ha, Ki-Tack Kim, Dong-Gune Chang, Hyung-Youl Park, Eun-Ji Yoon, Sang-Il Kim

**Affiliations:** 1 Department of Orthopedic Surgery, Seoul St. Mary’s Hospital, The Catholic University of Korea, 222, Banpo-daero, Seocho-gu, Seoul, 06591 Korea; 2 Department of Orthopedic Surgery, Kyung-Hee University Hospital at Gangdong, Kyung-Hee University, Seoul, Korea; 3 Department of Orthopedic Surgery, Sanggye Paik Hospital, Inje University, Seoul, Korea; 4 Department of Orthopedic Surgery, Eunpyeong St. Mary’s Hospital, The Catholic University of Korea, Seoul, Korea

**Keywords:** Medical research, Risk factors

## Abstract

During lateral lumbar interbody fusion (LLIF), unintended intraoperative endplate injury (IEPI) can occur and thereafter lead cage subsidence. The aim of this study was to investigate the incidence of IEPI during LLIF, and its predisposing factors. A retrospective review was conducted on consecutive patients (n = 186; mean age, 70.0 ± 7.6 years) who underwent LLIF at 372 levels. Patient’s demographic and surgical data were compared between patients with and without IEPI. Also, the radiographic data of each level were compared between intact and IEPI segments. IEPI was identified at 76 levels (20.4%) in 65 patients. The incidences of IEPI at every 100 consecutive segments were not different. When 372 segments were analyzed independently, sagittal disc angle (DA) in the extended position (4.3° ± 3.6° at IEPI segments vs. 6.4° ± 4.0° at intact segments), the difference between sagittal DA in the extended position and cage angle (− 2.2° ± 4.0° vs. 0.0° ± 3.9°), and the difference between preoperative disc height and cage height (− 5.4 mm ± 2.4 mm vs. − 4.7 mm ± 2.0 mm) were different significantly. Also, endplate sclerosis was more common at intact segments than IEPI segments (33.2% vs. 17.3%). Multivariate analysis showed that male sex (odds ratio [OR] 0.160; 95% confidence interval [CI] 0.036–0.704), endplate sclerosis (OR 3.307; 95% CI 1.450–8.480), and sagittal DA in the extended position (OR 0.674; 95% CI 0.541–0.840) were significant associated factors for IEPI. IEPI was correlated not with surgeon’s experience, but with patient factors, such as sex, preoperative disc angle, and endplate sclerosis. Careful surgical procedures should be employed for patients with these predisposing factors.

## Introduction

Lumbar interbody fusion (LIF) has been widely used to treat degenerative diseases presenting with low back pain and neurological symptoms. LIF may increase fusion rate and restore ideal segmental lordosis. Traditionally, LIF has been performed via several open approaches; anterior, posterior, or transforaminal. Among these options, anterior LIF (ALIF) has some advantages. It can provide greater bone-graft contact surface and the possibility to insert more lordotic cages, improving sagittal balance^1–4^. However, some pitfalls of conventional ALIF exit: great vessel injury, abdominal organ injury, and incisional hernia^5^.

Several minimally invasive spine surgical techniques have been developed to achieve better clinical outcomes and reduce postoperative complications compared to conventional open procedures. Minimally invasive surgery (MIS) has been shown to reduce postoperative pain; perioperative complications including hospital stay, blood loss, and need for analgesics; and lead to earlier recovery compared to conventional open techniques^6–8^. Recently, MIS-lateral LIF (LLIF) has gained popularity because it can provide similar effects as ALIF using tubular retractors. It can be performed by two approaches, (1) extreme LIF (XLIF), which accesses the intervertebral disc via transpsoas and (2) oblique LIF (OLIF), which is accessed via the oblique corridor between the aorta and the psoas muscle. The processes, in common, consist of discectomy, endplate preparation, and cage placement.

However, these LLIF procedures can result in several perioperative complications, including nerve injury, vascular injury or endplate injury^9–11^. Among these, endplate injury often occurs during endplate preparation and cage placement. Once it occurs, it can result in cage subsidence, leading to the loss of segmental lordosis and foraminal height. This type of endplate injury has features different from spontaneous cage subsidence in the postoperative course. Because the former is considered iatrogenic, careful surgical skills are indispensable. However, patient-related factors may also affect the development of intraoperative endplate injury (IEPI)^12^. The purpose of this study was to investigate the incidence of IEPI and identify its risk factors in our consecutive MIS-LLIF series.

## Results

A total of 186 patients were eligible for inclusion in this study. The mean age at the time of surgery was 70.0 ± 7.6 years (range 31–85) and 79.6% (148/186) were female. In those patients, 372 levels were treated using MIS-LLIF. The most common segment was L2-3 (170 segments, 45.7%), followed by L3-4 (130 segments, 34.9%), L1-2 (52 segments, 14.0%), and L4-5 (20 segments, 5.4%). Forty-two patients underwent single-level LLIF and 144 patients underwent multiple level LLIF. The patient demographic details are shown in Table 1.

**Table 1:** Patient demographic and surgical data.

Parameter	Value
Age (years)	70.0 ± 7.6 (31–85)
Male:female	38:148
**Mean BMD at lumbar spine**	
Bone density (g/m^2^)	1.111 ± 0.205
T-score	− 1.0 ± 1.5
Mean BMI (kg/m^2^)	25.1 ± 3.6
Total number of LLIF levels	372 (2.0 per patient)
No. of patients with single level : multiple levels	42:144
**LLIF level**	
L1-2	52
L2-3	170
L3-4	130
L4-5	20

*BMD* bone mineral density, *BMI* body mass index, *LLIF* lateral lumbar interbody fusion.

### Incidence of IEPI

**Figure 1:**
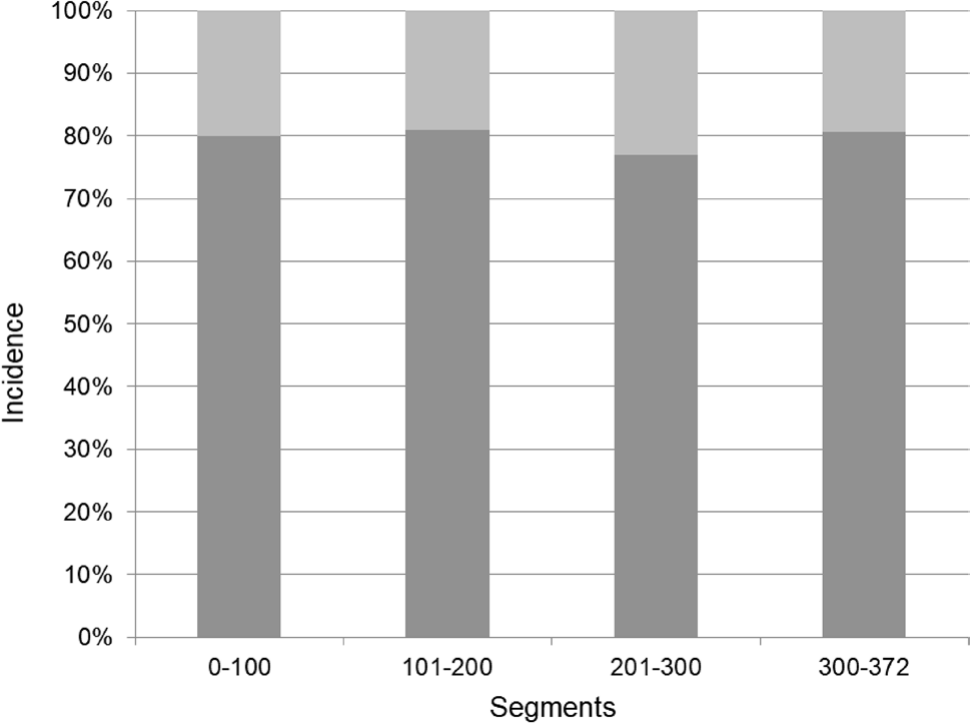
The incidence of intraoperative endplate injury at every consecutive 100 segments shows no significant difference.

The interobserver intra-class coefficients (ICC) for the measurement of IEPI was 0.842. The intraobserver ICC were 0.933 and 0.886 for each examiner. IEPI was identified at 76 levels (20.4%) in 65 patients. Unilateral endplate injury, indicated by damage of either endplate in an intervertebral disc, was noted at 67 levels and bilateral injury, indicated by damage of both endplates in an intervertebral disc, was noted at nine levels. Injury at the endplate cranial and caudal to the disc was noted at 33 and 52 levels, respectively (Table 2). The incidence of IEPI at each level ranged from 19.4% (33/170) to 35% (7/20). However, significant differences between the incidence at each segment were not found (Table 2). The type of IEPI had no association with the disc level. The incidence of IEPI every 100 segments is shown in Fig. 1. There was no significant difference between the incidence in each group.

**Table 2:** The numbers of segments with intraoperative endplate injury (IEPI) according to the location and disc level.

No. of segments with IEPI	76	
**By location**		
Unilateral endplate	67	
Bilateral endplates	9	
Endplate cranial to disc	33	
Endplate caudal to disc	52	

By level		P^#^
L1-2	11/52 (21.2%)	0.633
L2-3	33/170 (19.4%)	
L3-4	26/130 (20.0%)	
L4-5	6/20 (30.0%)	

^#^Linear-by-linear association test was performed.

### Characteristics of IEPI segments and risk factors

Age, BMI, BMD of the lumbar spine, and whether single- or multiple-level LLIF were not different between the patients with IEPI and the patients without IEPI (Table 3). Sex distribution was significantly different between the two groups (Table 3). When 372 LLIF segments were analyzed independently, coronal DA and sagittal DA in the neural and flexed positions were not different between the segments with and without IEPI (Table 4). The degree of facet arthrosis and disc height were not different between the two groups. However, sagittal DA in the extended position was significantly smaller at the IEPI segments than the intact segments (4.3° ± 3.6° vs. 6.4° ± 4.0°, *P* = 0.001). The difference between sagittal DAs in the extended position and cage angle was significantly different (− 2.2° ± 4.0° at IEPI segments vs. 0.0° ± 3.9° at intact segments, *P* < 0.001). Also, the difference between the preoperative disc height and the cage height was greater in the IEPI segments than in the intact segments (− 5.4 mm ± 2.4 mm vs. − 4.7 mm ± 2.0 mm, *P* = 0.042). The presence of endplate sclerosis was more frequent at the intact segments compared to the IEPI segments (33.2% [67/296] vs. 17.3% [9/76], *P* = 0.028). The incidence of IEPI did not differ significantly according to the cage angle and height (data not shown).

**Table 3:** Comparison of the patient data between the IEPI group and no-IEPI group.

	Patients without IEPI (n = 121)	Patients with IEPI (n = 65)	P
Age (years)	70.0 ± 7.6	69.9 ± 7.7	0.898^#^
Female (%)	71.9%	93.8%	< 0.001*
BMI (kg/m^2^)	25.3 ± 4.0	24.4 ± 4.8	0.111^#^
Mean T-score (lumbar spine)	− 0.9 ± 1.7	− 1.2 ± 1.6	0.084^#^
Single-level surgery (%)	14.3%	21.5%	0.301*

*BMI* body mass index, *NS* not significant.

^#^Student’s t-test was performed.

*Chi-squared test was performed.

**Table 4:** Comparison of demographic and radiographic data between intact segments and intraoperative endplate injury (IEPI) segments.

	Intact segments (n = 296)	IEPI segments (n = 76)	P
Age (years)	69.5 ± 8.0	71.8 ± 5.6	NS^#^
Female (%)	224 (75.7)	72 (94.7)	< 0.001*
BMD (g/cm^2^)	1.189 ± 0.250	1.136 ± 0.237	NS^#^
**Disc angle (°)**			
Coronal	1.1 ± 5.2	1.3 ± 5.4	NS^#^
Sagittal (neutral)	3.5 ± 4.5	2.2 ± 3.7	NS^#^
Sagittal (flexion)	0.3 ± 4.4	− 1.0 ± 4.2	NS^#^
Sagittal (extension)	6.4 ± 4.0	4.3 ± 3.6	0.001^#^
**Disc height (mm)**			
Anterior	7.0 ± 3.3	6.3 ± 3.2	NS^#^
Posterior	4.7 ± 1.9	4.9 ± 2.2	NS^#^
Mean	5.9 ± 2.2	5.5 ± 2.6	NS^#^
Disc sagittal angle (extension)—cage angle (°)	0.0 ± 3.9	− 2.2 ± 4.0	< 0.001^#^
Mean disc height—cage height (mm)	− 4.7 ± 2.0	− 5.4 ± 2.4	0.042^#^
Endplate sclerosis (%)	67 (33.2)	9 (17.3)	0.028*

*BMD* bone mineral density, *NS* not significant.

^#^Student’s t-test was performed.

*Chi-squared test was performed.

Multivariate logistic regression analysis revealed that sex, sagittal DA in the extended position, and endplate sclerosis were risk factors for IEPI (Table 5). Male sex (odds ratio [OR] 0.160; 95% confidence interval [CI] 0.036–0.704; *P* = 0.015) and endplate sclerosis (OR 3.307; 95% CI 1.450–8.480; *P* = 0.005) were negatively associated with the development of IEPI. Smaller sagittal DA in the extended position was a risk factor for IEPI (OR 0.674; 95% CI 0.541–0.840; *P* < 0.001).

**Table 5:** Risk factor analysis for intraoperative endplate injury (IEPI).

	Univariate		Multivariate	
	OR (95% CI)	P	OR (95% CI)	P
Sex (male)	0.173 (0.061–0.490)	0.001	0.160 (0.036–0.704)	0.015
Age	1.046 (0.999–1.095)	0.058	1.038 (0.982–1.097)	0.187
**Disc angle**				
Sagittal (neutral)	0.936 (0.868–1.009)	0.084	–	1.000
Sagittal (extension)	0.859 (0.783–0.943)	0.001	0.674 (0.541–0.840)	< 0.001
Sagittal (flexion)	0.935 (0.868–1.007)	0.075	0.946 (0.806–1.111)	0.500
Sagittal disc angle (neutral)—cage angle	0.925 (0.858–0.996)	0.040	–	1.000
Disc sagittal angle (extension)—cage angle	0.854 (0.781–0.934)	0.001	–	1.000
Mean disc height—cage height	0.854 (0.732–0.995)	0.044	0.903 (0.727–1.123)	0.360
Endplate sclerosis	2.371 (1.091–5.151)	0.029	3.307 (1.450–8.480)	0.005

Logistic regression analysis was performed.

## Discussion

The incidence of IEPI was 20.4% (76/372), which was higher than that in the previous reports. This might have resulted from our definition of IEPI, which was as at least 1 mm of cage settling, compared to the study by Satake et al.^12^ that defined IEPI was defined as at least 2 mm of cage settling. Vertebral endplate thickness has been reported to range from 0.35 to 1.03 mm^13–17^. Therefore, the authors decided to use at least 1 mm as the criteria for IEPI in this study.

The vertebral endplate is a thin cortical bone located at the cranial and caudal surfaces of the vertebral bodies. In their histologic study, Hou et al.^18^ showed that the endplate was not genuine cortical bone but a porous structure with the involvement of trabeculae. The significance of the endplate had been already demonstrated in many reports. Removal of the endplate can significantly decrease the structural properties of the lumbar vertebral bodies^18–21^. Interbody cages in the lumbar spine are commonly used to increase mechanical stability and promote fusion, however, lumbar vertebrae with endplate damage have a higher risk of cage subsidence.

The factors associated with cage subsidence following intervertebral fusions have been reported to involve BMD^22^, cage geometry^23,24^, cage material^25^, cage location^26–29^, and the use of osteobiologics^30^. Cage subsidence is thought to result from biological remodeling at the cage-bone interface in a chronic fashion^31^. In contrast, IEPI develops in an acute fashion during the surgical procedure and the development of IEPI may have the different pathomechanisms and risk factors. There were little studies about IEPI after MIS-LLIF. Two risk factors for IEPI after MIS-LLIF had been reported in the study by Satake et al.^12^: reduced BMD and cage height.

Although our results that female sex was a significant risk factor for IEPI in this study could suggest a possible correlation between BMD and IEPI, the direct measurement of BMD in each vertebra was not correlated with IEPI, unlike the previous report^12^. BMD, which is usually assessed by DEXA, shows trabecular bone quality. Hou et al.^26^ and Patel et al.^32^ conducted biomechanical tests on human cadaveric lumbar vertebrae and their results indicated that reduced BMD was positively associated with the failure load of the endplate, which ranged from 20 to 50%. They concluded that the lumbar vertebrae with reduced BMD had a higher risk of cage subsidence^26,32^. However, IEPI actually means cortical bone injury. Thus, the authors concluded that IEPI might be affected by cortical bone strength. Some parameters for cortical bone status of the endplate have been reported in previous studies. The endplate cranial to the intervertebral disc was thicker and had a higher density than the caudal one^13–17,33,34^. Based on this biomechanical property, IEPI could be expected to occur at the weak endplate. Our findings that the endplate injury was more common at the caudal endplate and one of the associated factors was endplate sclerosis (as a protective factor) supported the previous data.

Also, the characteristics of the intervertebral disc should be considered as factors affecting the development of IEPI. Previous reports showed that over-distraction of the intervertebral height by a tall cage could damage the endplate either intraoperatively or postoperatively^12,34^. However, there was no effect of segmental disc height and its difference from cage height on the development of IEPI. In the current study, the degree of facet arthrosis was not associated with the development of IEPI. However, a smaller sagittal disc angle in the extended position was found to be positively correlated with IEPI. It is plausible that the extent of disc motion could affect the development of IEPI; the more mobile disc, the less IEPI, and vice versa. We believe our results support this hypothesis. When planning LLIF followed by posterior spinal fixation in patients with less mobile discs, a different surgical strategy, for example, 3-stage surgery (posterior facetectomy-to-LLIF-to-posterior fixation) should be considered.

IEPI was considered somewhat iatrogenic in the previous reports. However, the conclusions were not an evidence-based, but empirical conclusions^10,12^. To the best of our knowledge, no study has analyzed the learning curve for IEPI after MIS-LLIF. In our study, there was no difference in the incidences of IEPI during MIS-LLIF at every 100 discs. This suggests that the iatrogenic factor for IEPI was minimal or none.

There were some limitations in our study. First, this was a retrospective study. Therefore, the factor of surgeon’s caution in osteoporotic patients could not be considered. Second, the radiographic measurement of the endplate injury was not verified because it could be missed on plain radiographs, especially in patients with scoliosis. Third, we did not evaluate the radiographic findings and clinical prognosis during the late period. IEPI was a radiographic finding and its clinical significance was not analyzed. However, some patients with IEPI experienced progressive cage subsidence and even vertebral body fracture. We are preparing the next article about clinical outcomes of IEPI. Fourth, the quantification of IEPI was not included in this study. The authors defined IEPI as > 1 mm of endplate damage. However, the surgical prognosis could be different according to the severity of the IEPI.

In conclusion, this study showed that the development of IEPI after MIS-LLIF was significantly correlated with some patient-related factors, including gender, sagittal disc angle in the extended position, and endplate sclerosis, whereas the surgeon’s experience did not affect the development of IEPI. Therefore, patients who have these risk factors are at risk of IEPI after MIS-LLIF. Thorough preoperative evaluation is needed to avoid IEPI when considering MIS-LLIF surgery and careful surgical procedures should be performed in patients with an elevated risk.

## Methods

### Patients

This retrospective study was approved by The Catholic University of Korea Catholic Medical Center’s Institutional Review Board before study initiation and all methods were performed in accordance with the relevant guidelines (approval no. KC20RISI0169). Informed consents were waived by The Catholic University of Korea Catholic Medical Center’s Institutional Review Boar because of the retrospective study design. All consecutive patients who underwent MIS-LLIF for degenerative lumbar disc diseases (from L1-2 to L4-5) between May 2012 and December 2017 were reviewed and the operative data in the medical records were investigated. To minimize bias, patients who underwent operations by surgeons other than the single senior author (KYH) were excluded. The patients included in this study were the first 186 patients who underwent MIS-LLIF performed by this surgeon. Clinical data, including age, sex, body mass index (BMI), and bone mineral density (BMD) were reviewed in the medical records. BMD at the L1-4 levels of the posteroanterior spine was measured using dual-energy X-ray absorptiometry (DEXA) bone densitometry (Lunar Prodigy Advance, GE Healthcare, Waukesha, WI, USA). T-scores of the lumbar spine (L1-4) were recorded and BMD in each vertebra was recorded as g/m^2^.

### Surgical procedures

MIS-LLIF was performed, according to XLIF manner, by splitting the psoas muscle using tubular retractors and intraoperative neuromonitoring^5^. All procedures were performed in a right lateral decubitus position with the hip and knee joints flexed. A surgical incision was made through the skin after identification of the position of the target disc using a C-arm. A single 5-cm skin incision was made in patients who underwent LLIF in one or two segments, whereas two separate incisions were made in patients who underwent LLIF in three or more segments. The retroperitoneal space was reached via blunt dissection and tubular retractors were placed onto the disc. After that, removal of the disc materials and endplate preparation was conducted. For endplate preparation, a Cobb elevator and ring curette were used. None of the shavers was used at any step. The cage size was determined using trial cages and then the real cage was inserted with two containment blades. The dimensions of each cage were recorded in the medical charts. All procedures were performed under fluoroscopic guidance. Poly-etherether-ketone (PEEK) cages (Clydesdale, Medtronic Sofamor Danek, Memphis, TN, USA) were used in all patients. After anterior surgery, posterior fusion with pedicle screws was performed in a single or staged manner.

### Radiographic measurements

**Figure 2:**
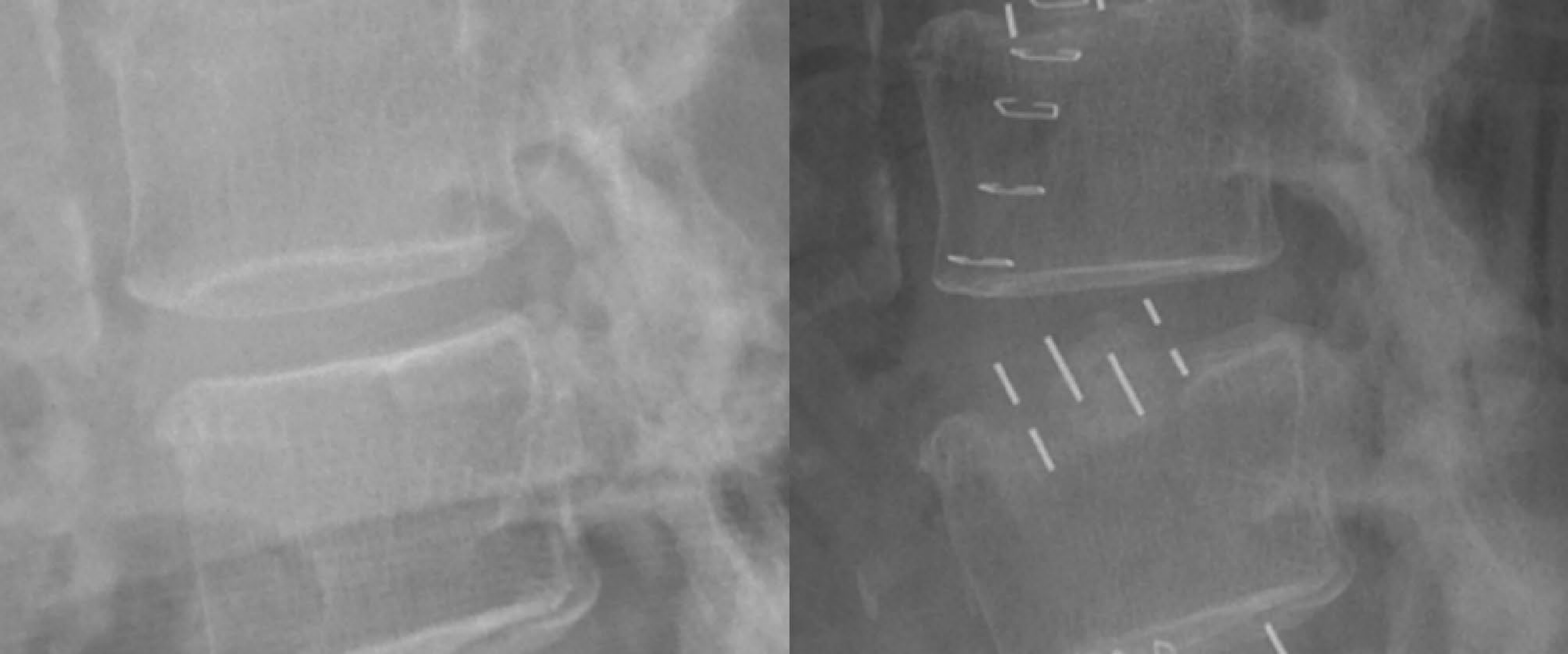
Intraoperative endplate injury during lateral lumbar interbody fusion can be identified by comparison between the preoperative (left) and postoperative (right) X-rays.

IEPI was identified on the immediate postoperative lateral X-ray compared to the preoperative lateral X-ray. It was defined as cage sinking of more than 1 mm from the bony endplate (Fig. 2). Two spine fellows, who had not been participate in the surgery, independently measured the extent of endplate injury and the same measurements were repeated by these two examiners. The average values were obtained and used in the definition of IEPI. IEPI was classified into two groups based on involvement: unilateral or bilateral, superior or inferior. The profiles of the inserted cage, such as its height and lordotic angle, were recorded. The following parameters of each intervertebral disc were measured: (1) segmental disc angle (DA) on the sagittal plane in the neural, flexed, and extended positions and (2) disc height (DH) in the anterior and posterior corners. We calculated the differences in their height and angle between the disc and the cage. Because endplate sclerosis could affect endplate injury, the existence of sclerosis was investigated. Four grades of facet joint arthrosis were identified on the computed tomography (CT) and magnetic resonance imaging (MRI) using the criteria proposed by Weishaupt et al.^35^.

### Overall incidence of intraoperative endplate injury and its distribution according to experience

The incidence of IEPI in each segment (from L1–2 to L4–5) was calculated. Because the development of IEPI could be affected by surgical skills, the authors divided the cohort into four arbitrary groups, one every 100 segments, and analyzed the IEPI incidence of each group to evaluate learning-curve effects.

### Statistical analysis

SPSS 21.0 (SPSS Inc., Chicago, IL, USA) was used for all statistical analyses. The demographic, surgical, and radiographic data were compared between the IEPI group and the no-IEPI group. The Chi-squared test was used for categorical data and the Student’s t-test was used for continuous data. To identify the risk factors for IEPI, univariate analysis for each parameter of demographic, surgical, and radiographic data was performed using logistic regression analysis. Parameters with a *P*-value ≤ 0.10 were included in multivariate logistic regression analysis. Inter- and intraobserver reliability for the measurement of IEPI were analyzed using intra-class coefficients (ICC). Statistical significance was considered at *P* < 0.05.
